# Mitochondrial Calcium uniporters are essential for meiotic progression in mouse oocytes by controlling Ca^2+^ entry

**DOI:** 10.1111/cpr.13127

**Published:** 2021-09-21

**Authors:** Lu yao Zhang, Meng Lin, Zhuan Qingrui, Wang Zichuan, Li Junjin, Liu Kexiong, Fu Xiangwei, Hou Yunpeng

**Affiliations:** ^1^ State Key Laboratories of Agrobiotechnology College of Biological Sciences China Agricultural University Beijing China; ^2^ Key Laboratory of Animal Genetics, Breeding and Reproduction College of Animal Science and Technology China Agricultural University Beijing China; ^3^ State Key Laboratory of Sheep Genetic Improvement and Healthy Breeding Xinjiang Academy of Agricultural and Reclamation Sciences Shihezi China

**Keywords:** AMPK, meiosis, mitochondrial Ca^2+^, mitochondrial function, oocytes

## Abstract

**Objectives:**

The alteration of bioenergetics by oocytes in response to the demands of various biological processes plays a critical role in maintaining normal cellular physiology. However, little is known about the association between energy sensing and energy production with energy‐dependent cellular processes like meiosis.

**Materials and methods:**

We demonstrated that cell cycle‐dependent mitochondrial Ca^2+^ connects energy sensing to mitochondrial activity in meiosis progression within mouse oocytes. Further, we established a model in mouse oocytes using siRNA knockdowns that target mitochondrial calcium uniporters (MCUs) in order to inhibit mitochondrial Ca^2+^ concentrations.

**Results:**

Decreased numbers of oocytes successfully progressed to the germinal vesicle stage and extruded the first polar body during in vitro culture after inhibition, while spindle checkpoint‐dependent meiosis was also delayed. Mitochondrial Ca^2+^ levels changed, and this was followed by altered mitochondrial masses and ATP levels within oocytes during the entirety of meiosis progression. Abnormal mitochondrial Ca^2+^ concentrations in oocytes then hindered meiotic progress and activated AMP‐activated protein kinase (AMPK) signalling that is associated with gene expression.

**Conclusions:**

These data provide new insight into the protective role that MCU‐dependent mitochondrial Ca^2+^ signalling plays in meiotic progress, in addition to demonstrating a new mechanism of mitochondrial energy regulation by AMPK signalling that influences meiotic maturation.

## Graphical abstract

When KD‐MCU occurs in oocytes due to impaired mitochondrial function, cytosolic ATP levels decline. The energy sensor AMPK is activated and phosphorylated in response to the increased energetic stress. Excessive activation of AMPK results in adverse effects on the resumption of meiosis. In addition, microtubule dynamics and tension establishment cannot efficiently be achieved, leading to decreased meiotic progression. These observations all implicate MCU as being critical for meiotic progression.

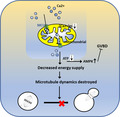

## 1 INTRODUCTION

Mitochondria are particularly important organelles in germ cells whose functions exhibit dual impacts on energy status. Mitochondria produce adenosine triphosphate (ATP) and reactive oxygen species that are natural by‐products of oxidative phosphorylation.^1^ Both of these compounds regulate and control biological processes. Previous studies have shown that mitochondria exhibit typical maternal hereditary characteristics, wherein maternally derived mitochondria comprise the entire population in offspring cells. Mitochondrial damage in oocytes due to obesity, diabetes and ageing reduces female reproductive capacity.^2^ Interestingly, we previously observed that maternal metabolic syndromes induce metabolic alterations in oocyte mitochondria.^3^ Calcium (Ca^2+^) acts as a secondary messenger that regulates various biological processes including differentiation, apoptosis and cell division,^4^ while calcium signalling plays vital roles in cell cycle regulation.^5^ Numerous studies have shown that there is a rapid rise in Ca^2+^ levels in the cytoplasm during mitosis and oocyte maturation.^6^ Oocytes spend a considerable amount of time having entered meiosis and are arrested at the first meiotic prophase.^7^ In preparation for fertilization, mammalian oocytes undergo a process that causes dramatic increases in cytosol Ca^2+^ levels.^8^ Thus, these observations indicate that Ca^2+^ transiently promotes meiotic progression.

Increased cytosolic Ca^2+^ levels lead to rapid Ca^2+^ uptake by mitochondria. Numerous studies have shown that mitochondrial calcium uniporters (MCUs) are unique channels that control calcium influxes.^9^, ^10^ Further, mitochondrial Ca^2+^ levels promote the activities of the mitochondrial electron transport chain and the tricarboxylic acid (TCA) cycle, leading to ATP production.^11^, ^12^ Indeed, mitochondria are the primary sources of energy for ATP synthesis, which is required for numerous biological processes.^13^ Importantly, abnormal accumulation of mitochondrial Ca^2+^ can lead to mitochondrial dysfunction, decreased ATP production and other problems.^14^ Consequently, ATP synthesis is an indicator of mitochondrial function, further highlighting the indispensable relationship between energy sources and mitochondrial function.^15^ Meiotic progression demands high energy inputs to drive DNA synthesis and chromosome segregation, which are critical for oocyte maturation.^15^, ^16^ Therefore, a close association exists between mitochondrial Ca^2+^ signalling and meiosis, although the underlying mechanisms are still unknown.

AMP‐activated protein kinase (AMPK) signalling is a crucial cellular energy sensory mechanism. AMPK is a unique protein kinase that is regulated by the ATP/energy status and helps re‐balance ATP production.^14^ The kinase activity of AMPK complexes is instantaneously increased by acutely altered AMP levels, suggesting AMPK activation is due to ATP consumption and AMP accumulation.^17^ ATP is mainly synthesized in oocytes via the mitochondria, which are the energy factories of cells. In this study, we investigated the key role of MCU‐mediated mitochondrial Ca^2+^ homeostasis in maintaining energy homeostasis during meiosis. In response to the low energy status during meiosis, the energy sensor AMPK phosphorylates and prevents meiotic maturation. Our results demonstrate a critical role for MCU‐dependent mitochondrial Ca^2+^ signalling that connects energy sensing to proper meiotic progression and AMPK phosphorylation.

## 2 METHODS AND MATERIALS

### 2.1 Ethics statement

Unless otherwise stated, all chemicals and medicines were purchased from the Sigma Chemical Co. (St Louis). Three‐week‐old CD‐1 ® (ICR) female mice were purchased from the Beijing Vital River Experimental Animals Centre and were housed at the department of animal experiments under standard housing conditions. The Laboratory Animal Care and Use Committee of the Institute of Zoology approved this study (AW01040202‐1).

### 2.2 Animal experiments

Mice were kept under 12‐h/12‐h light–dark cycles in a dedicated pathogen‐free environment at the Central Animal Laboratory of the Institute of Zoology. All procedures were performed with the approval of the Institutional Animal Care and Use Committee of China Agricultural University (AW01040202‐1).

### 2.3 Oocyte collection and culture

Germinal vesicle (GV) stage oocytes were collected from the 3‐week‐old ICR mice. 5 IU of pregnant mare serum gonadotropin (PMSG, Ningbo second hormone factory) was injected into the mice 46–48 h before all experiments. GV‐stage oocytes were released from the fully grown follicles into pre‐warmed M2 medium supplemented with 2.5 μM milrinone, and cumulus cells were removed by repeated pipetting. After microinjection or another specific treatment, oocytes were thoroughly washed with DPBS and cultured in MI6 medium under mineral oil at 37°C in a 5% CO_2_ atmosphere incubator during the GV to MII stages.

### 2.4 Measuring mitochondrial Ca^2+^ ([Calcium]_m_)

[Calcium]_m_ levels were measured using Rhod‐2AM (Invitrogen/Molecular Probes, Carlsbad) according to the manufacturer's instructions. Zona pellucida was removed by pronase E. The oocytes were then stained with 5 μM Rhod‐2AM for 30 min in maturation medium and thoroughly washed with DPBS, followed by incubation in maturation medium‐free Rhod‐2AM at 37°C under a 5% CO_2_ atmosphere for 30 min. Cells were subsequently observed with confocal laser scanning microscopy (Nikon A1R) and quantified using a NIS‐Elements AR (Nikon Instruments).

### 2.5 Measuring cytosolic Ca^2+^ ([Calcium]_i_)

Cytosolic Ca^2+^ levels were assessed using Flou‐3 AM (Invitrogen/Molecular Probes, Carlsbad). First, zona pellucid was enzymatically removed by 0.5%pronase 37°C for 5 min. The oocytes were then processed in maturation medium with 5 μM Flou‐3 AM for 40 min and washed three times by DPBS. Subsequently, they were analysed using a confocal laser scanning microscope (Nikon A1R) and quantitatively processed using NIS‐Elements AR (Nikon Instruments).

### 2.6 siRNA microinjection

Small interference RNAs (siRNA) for MCU (CCAAAGAGACCUAATTUUAG GAGGUCUCUCUUUGGTT) and

Mad2 (GGACUCACCUUGCUUACAATTUUGUAAGCAAGGUGAGUCCTT) (Gene Pharma) or siRNA‐negative controls were microinjected (5 μM) into fully grown immature oocytes with an Eppendorf microinjection instrument (Hamburg) and allowed to incubate for 30 min. Oocytes were arrested in the GV stage in MI6 medium (Sigma‐Aldrich) supplemented with 2.5 μM milrinone for 20–24 h. The oocytes were then thoroughly washed with DPBS to resume meiosis.

### 2.7 Investigation of mitochondrial distributions

Mitochondrial distributions were evaluated using the mitochondrial reactive dye Mito‐tracker (Green) (Beyotime Institute of Biotechnology). Oocytes were placed in maturation medium with 5 μM Mito‐tracker (Green) for 20 min and thoroughly washed three times with DPBS. A confocal laser scanning microscope (Nikon A1R) was then used to investigate the oocytes, and an NIS‐Elements AR (Nikon Instruments) was used to quantify mitochondria.

### 2.8 Quantification of mitochondrial membrane potentials

A mitochondrial membrane potential assay kit (JC‐1 dye, Beyotime Institute of Biotechnology) was used to measure mitochondrial membrane potentials (Δφm). Oocytes were stained with a working solution containing 10 μM JC‐1 at 37.0°C in a 5% CO_2_ atmosphere for 20 min, after which they were washed with washing buffer (DPBS) to remove surface fluorescence, followed by observation with a fluorescence microscope (Olympus IX73). Red fluorescence corresponded to activated mitochondria (J‐aggregates), while green fluorescence corresponded to less activated mitochondria (J‐monomers), and the ratio is given as the Δφm value.

### 2.9 ATP content assays

The ATP content in each oocyte was measured with an Enhanced ATP Assay Kit, S0027 (Beyotime Institute of Biotechnology) according to the manufacturer's instructions. Different ATP standards were prepared, ranging from 0 to 40 pol ATP. Oocytes were then treated with 20 μM of lysis buffer within a 0.2‐ml RNA‐free centrifuge tube, and lysed cells were centrifuged for 5 min at 4°C and 12,000 *g*. All steps were conducted on ice unless otherwise stated. ATP detecting solution was then added to 96‐well plates and was left to sit at room temperature for 3–5 min. Standard solutions and ATP detection diluents were then added into each well. Samples were also added to each well, and the luminescence signals were immediately calculated with a luminometer (Infinite F200; Tecan). The ATP content of the samples was then calculated based on the standard curves. Total ATP levels were divided by the number of oocytes in each sample to calculate the mean ATP content per oocyte (pmol/oocyte).

### 2.10 Immunofluorescence

Mouse MI or MII oocytes were fixed in 4%(w/v) paraformaldehyde for 40 min at room temperature and washed three times (10 min each) in washing buffer (PBS containing 0.01%Triton X‐100 and 0.1% Tween‐20). The oocytes were then permeated in 1% Triton X‐100/PBS at room temperature for 1 h and washed three times (10 min each) in washing buffer. The oocytes were then blocked with blocking buffer (1% BSA /PHEM with 100 mM glycine) for 1 h at 37°C. The oocytes were incubated at 4°C overnight with anti‐α‐tubulin antibody (1:8000) diluted in blocking buffer. After washing three times with washing buffer for 10 min each, the oocytes were incubated at 37°C for 1 h with goat anti‐mouse‐FITC antibody (1:100 dilutions, CW Biotech) and washed four times in washing buffer for 10 min each. Finally, DNA was stained with 4’6‐diamidino‐2‐phenylindole (DAPI, Vector Laboratories Inc.). The oocytes were then expanded on glass slides and examined with confocal laser‐scanning microscopy (FLUOVIEW FV1000, Olympus) using the FLUOVIEW Viewer (Olympus). The excitation lasers were set at 488 nm, and emission channels of 520 nm were used for green fluorescence detection.

### 2.11 RNA sequencing

We performed expression profiling on pools of 30 denuded GV oocytes isolated per group. RNA was isolated using the RNeasy Micro Kit (Qiagen). cDNA was generated and amplified from 1.2 ng with the Nu‐Gen ovation RNA‐seq System V2 (Part no. 7102; Nu‐Gen). 50 ng of the resulting SPIA cDNA was fragmented, and sequencing libraries were prepared using Tru‐Seq DNA Sample Preparation Kit (low‐throughput protocol) (Part no. 15005180 Rev. C; Nu‐Gen). Libraries were pooled equimolarly and sequenced for 50 cycles on an Illumina Hi‐Seq 2000 instrument using RTA 1.13.48 for base calling. Demultiplexing and fast‐q generation were performed with bcl2fastq (bcl2fastq‐1.8.3).

### 2.12 RNA extraction, reverse transcription and quantitative PCR (Q‐PCR)

Total RNA was extracted from 40 GV, MI or MII oocytes using a RNeasy micro‐RNA isolation kit (Qiagen) following the manufacturer's instructions. Samples were treated with DNase I, and then Transcript‐Uni Cell was used for cDNA Synthesis. A Q‐PCR super‐mix was used for the assays (Trans Gen Biotech). RNA concentrations were measured using a Nanodrop 2000 Spectrophotometer (Biolab, Scoresby) at a wavelength of 260 nm. Samples for subsequent analyses were only used if their 260:280 nm absorbance ratios were >1.8. Primers for the published reference RNA sequences for real‐time Q‐PCR and RT‐PCR are listed in Table 1.Q‐PCR and RT‐PCR assays were performed with an ABI 7500 real‐time PCR instrument and a Fast 96‐well Thermal Cycler (Applied Biosystems), respectively. Three replicates were conducted for all assays. The relative expression of genes was calculated with the comparative threshold cycle (CT) method as 2^–ΔΔCT^. The primers used for the amplification assays are shown in Table 2.

**TABLE 1:** Effect of Compound C treatment on meiosis maturation recovery after MCU depletion

Groups	No. of oocytes culture	No. of oocytes GVBD (%, mean* ± SEM*)	No. of oocytes PB1 (%, mean* ± SEM*)
si‐control	145	139 (95.7667 *± *.71259)^a^	99 (88.4667* ± *1.38604)^a^
si‐MCU	116	74(63.8333* ± *1.67465)^c^	57 (61.9000* ± *4.30968)^b^
si‐MCU *+ *CompoundC	82	75 (90.2000* ± *.85440)^b^	48(81.3500* ± *.75000)^a^

Different superscript letters (a–c) indicate significant differences of measurements in the same column (*p* < 0.05); SEM, standard error of the mean.

**TABLE 2:** Oligonucleotide primer sequences used for quantitative real‐time PCR

Gene	Primer sequence (5’−3’)	Product size (bp)	GenBank accession number or reference
*MCU*	F: ACTCACCAGATGGCGTTCG R: CATGGCTTAGGAGGTCTCTCTT	129	NM_001033259
*mad2*	F: GTGGCCGAGTTTTTCTCATTTG R: AGGTGAGTCCATATTTCTGCACT	100	NM_019499
*gapdh*	F: AGGTCGGTGTGAACGGATTTG R: TGTAGACCATGTAGTTGAGGTCA	123	NM_008084
*β‐Actin*	F: GGCTGTATTCCCCTCCATCG R: CCAGTTGGTAACAATGCCATGT	154	NM_007393
*Plekhm2*	F: AAGGACCGAATCCTGGAGAAC R: TCTCTGCGGGTAAAATGGACC	206	NM_001033150
*Eci2*	F: TGCTCCTCTTACACGTTTCCG R: CGTTGACTGCGTAGAGCTTTTC	250	NM_011868
*Cd27*	F: CAGCTTCCCAACTCGACTGTC R: GCACCCAGGACGAAGATAAGAA	119	NM_001033126
*Cybb*	F: TGTGGTTGGGGCTGAATGTC R: CTGAGAAAGGAGAGCAGATTTCG	190	NM_007807
*Hilpda*	F: TGCTGGGCATCATGTTGACC R: TGACCCCTCGTGATCCAGG	109	NM_001190461
*Itgb3bp*	F: GAGCCCATTTTCTTCTCCCG R: GCAACACCATGAATCCATCCC	143	NM_026348
*Trim68*	F: TCCCAGAACTTGAGCTACAC R: AGACGGACCTTGTCTACAACA	104	NM_198012

### 2.13 Statistical analyses

All experiments were repeated at least three times. Data are presented as means *± SEM*, unless otherwise stated. Statistical comparisons were made with Student's t tests or one‐way ANOVA tests, where appropriate. A *p* < 0.05 was considered statistically significant.

## 3 RESULTS

### 3.1 Mitochondrial Ca^2+^ and ATP levels during oocyte maturation

**FIGURE 1:**
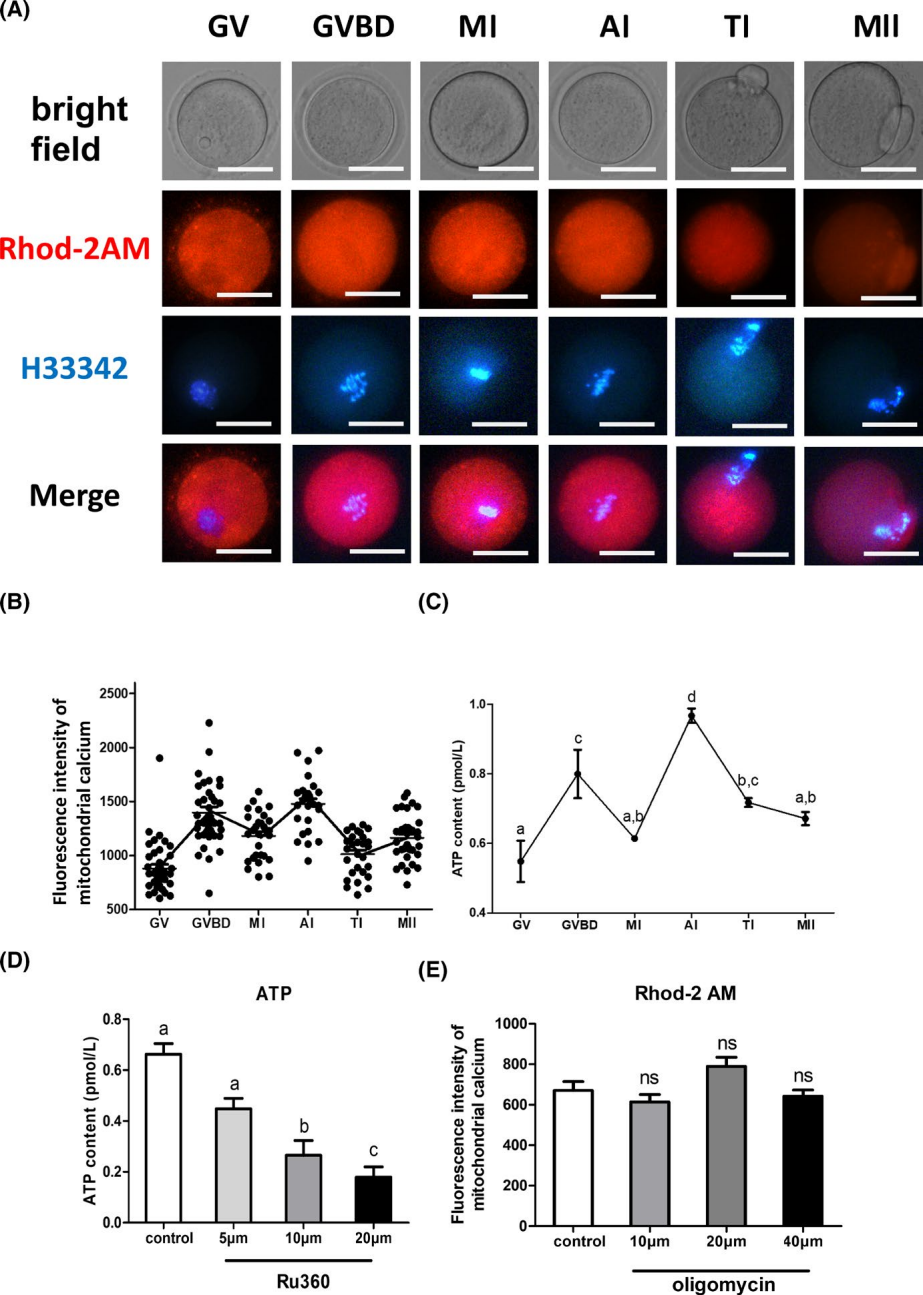
Mitochondrial calcium influx may play a key role during oocyte maturation and regulates ATP synthesis. (A) Representative images of Rhod‐2 AM fluorescence (red) in germinal vesicle (GV) stage oocytes and other meiosis stages. H33342 fluorescence is shown in blue. Scale bar: 50 μM. (B) Quantification of the relative levels of mitochondrial Ca^2+^ in GV‐stage oocytes and other meiosis stages (n = 50 for each group). (C) ATP concentrations (pM) were evaluated in germinal vesicle (GV) stage oocytes and in other meiosis stages (*n* = 30 for each group). (D) ATP concentrations (pM) were evaluated using different levels of Ru360 (5 μM, 10 μM and 20 μM) in germinal vesicle (GV) stage oocytes (*n* = 50 for each group). (E) Quantification of the relative levels of mitochondrial Ca^2+^ using different concentrations of oligomycin (10 μM, 20 μM and 40 μM) in GV‐stage oocytes (*n* = 50 for each group). Student's one‐way ANOVA tests were used to evaluate statistical differences in measurements. Different superscript letters (a–d) indicate significant differences within the same column of measurements (*p* < 0.05). Error bars show SEM

Mitochondrial Ca^2+^ and ATP levels were first evaluated during oocyte meiotic maturation by immunofluorescent staining and confocal microscopy. Quantitative analysis of mitochondrial Ca^2+^ and ATP contents indicated that changes in ATP content were consistent with mitochondrial calcium changes (Figure 1A–C). Thus, mitochondrial calcium influxes may play a key role during oocyte maturation. We consequently attempted to evaluate the upstream and downstream relationships between mitochondrial calcium and ATP during oocyte maturation. We postulated that mitochondrial Ca^2+^ had a direct effect on ATP production, based on our previous results.^18^ To address this possibility, we treated fully grown oocytes at the GV stage with different concentrations of Ru360 or oligomycin, which are inhibitors of mitochondrial Ca^2+^ influx or ATP synthesis. The low levels of mitochondrial Ca^2+^ resulted in lower ATP contents (Figure 1D–E), but low ATP contents had no effect on mitochondrial Ca^2+^ levels, suggesting that mitochondrial Ca^2+^ downregulated ATP production in mouse oocytes.

### 3.2 MITOCHONDRIAL CALCIUM UNIPORTERS is important for meiotic progression in mouse oocytes

**FIGURE 2:**
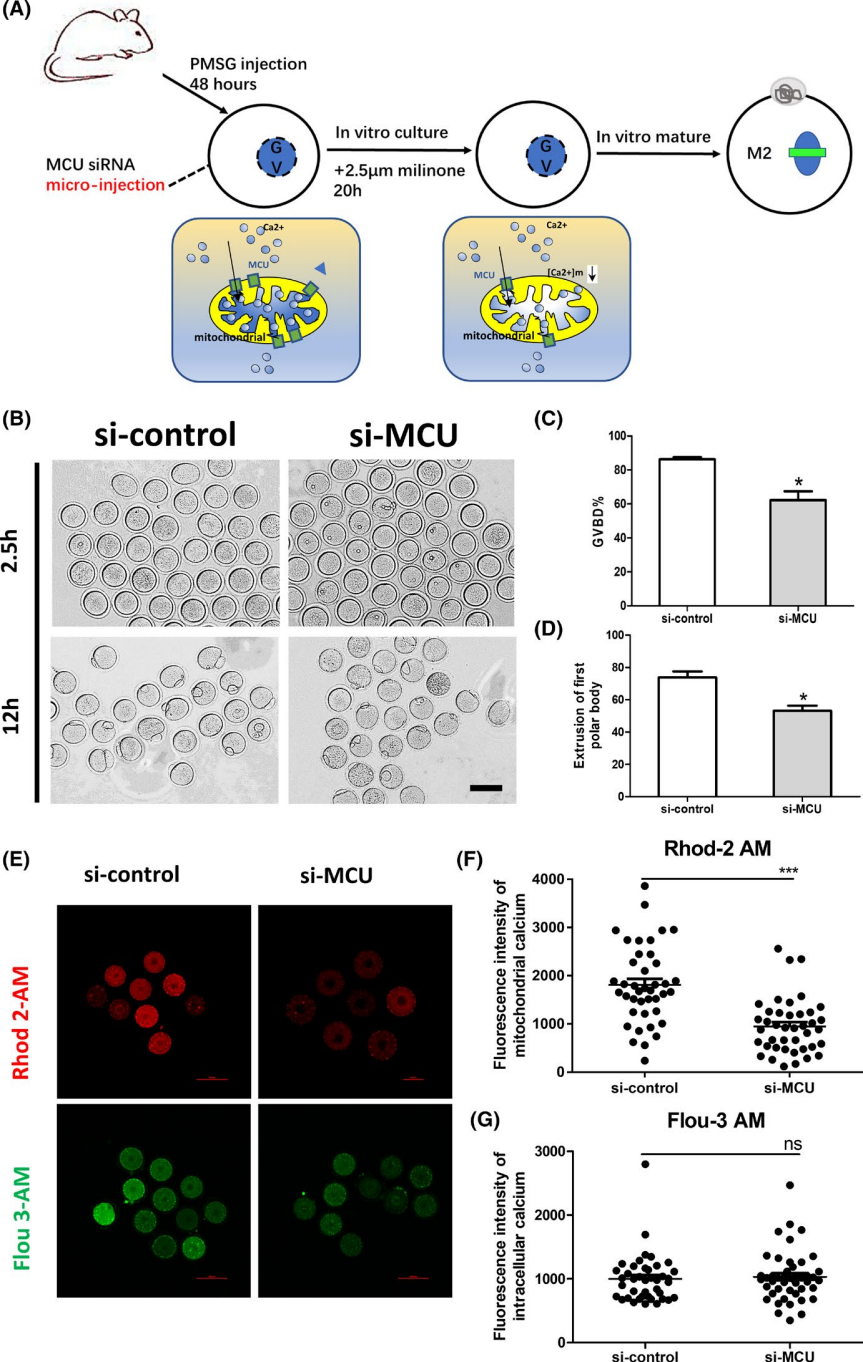
Oocyte maturation is impaired by MCU defects (A) Schematic showing the procedures for establishing defected MCU models by microinjection of siRNA sequences to knockdown MCU expression in mouse GV oocytes. (B) Representative images of germinal vesicle breakdown (GVBD) after 2.5 h and the first polar body (PB1) after 12 h for extrusion oocytes from the si‐control and si‐MCU groups. Scale bar: 50 μM. (C) The percentage of oocytes that successfully progressed to GVBD during *in vitro* culture for 2.5 h (*n* = 127 for si‐control, *n* = 134 for si‐MCU). (D) The percentage of oocytes that successfully extracted the first polar body during *in vitro* culture for 12 h (*n* = 109 for si‐control, *n* = 104 for si‐MCU). (E) Representative images of Rhod‐2 AM fluorescence (red) and Flou‐3 AM fluorescence (green) in germinal vesicle (GV) stage oocytes from the si‐control and si‐MCU groups. Scale bar: 50 μM. (F) Quantification of the relative levels of mitochondrial Ca^2+^ in GV‐stage oocytes from si‐control and si‐MCU (*n* = 41for each group). (G) Quantification of the relative levels of cytoplasmic Ca^2+^ in GV‐stage oocytes from si‐control and si‐MCU (*n* = 41 for si‐control, *n* = 46 for si‐MCU). A Student's *t* test was used for statistical analyses. *p < 0.05; **p < 0.01; ***p < 0.001; ns indicates nonsignificant (*p* > 0.05). Error bars show SEM

**FIGURE 3:**
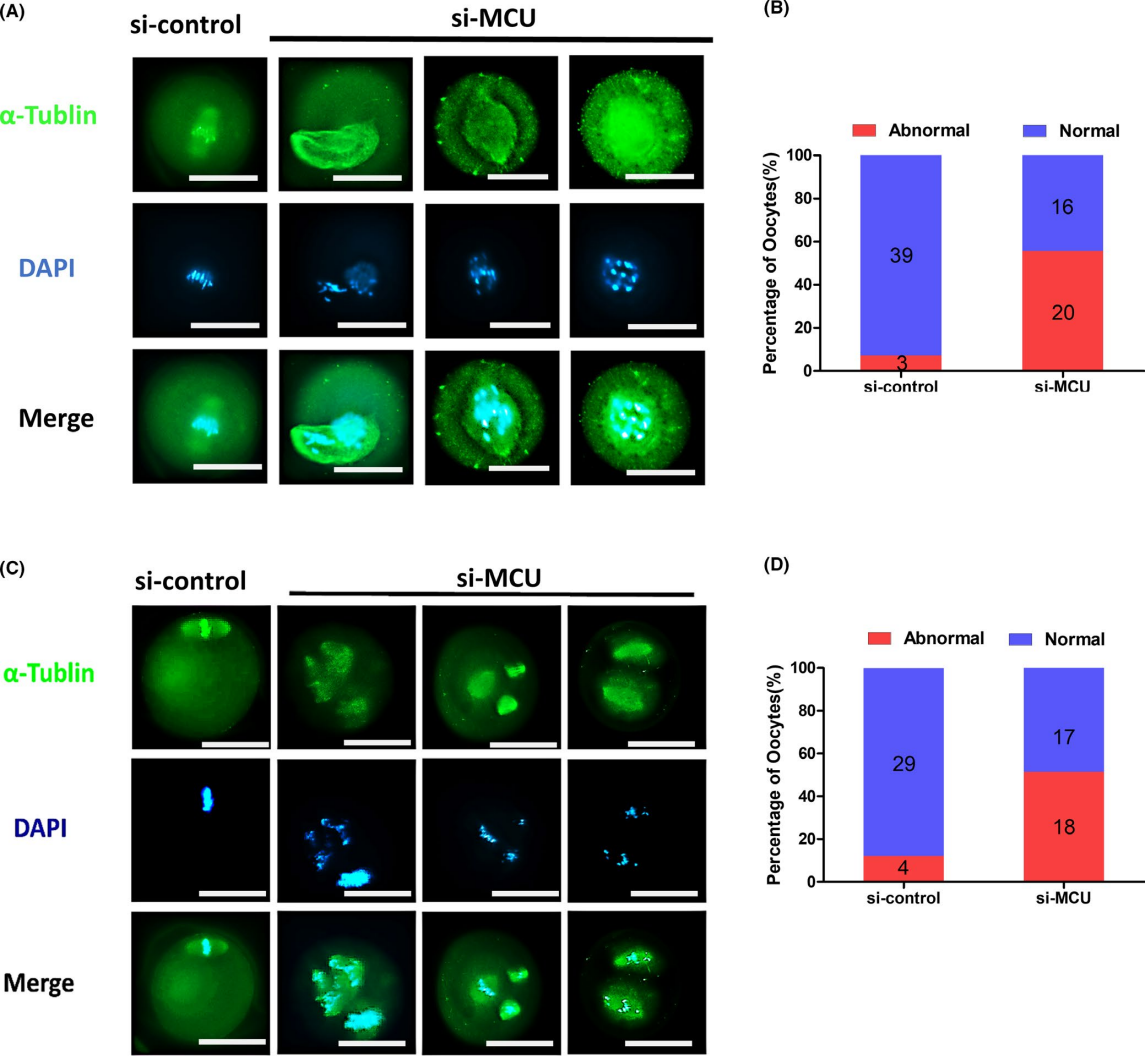
MCU is required for meiotic progress. (A) si‐control and si‐MCU MI oocytes were co‐stained with an α‐tubulin antibody to visualize spindles (green) and with DAPI (blue) to visualize chromosomes. (B) Quantification of si‐control and si‐MCU MI oocytes with abnormal spindles (si‐control: *n* = 42; si‐MCU: *n* = 36). (C) si‐control and si‐MCU M2 oocytes were co‐stained with an α‐tubulin antibody to visualize spindles (green) and with DAPI (blue) to visualize chromosomes. (D) Quantification of si‐control and si‐MCU M2 oocytes with abnormal spindles (si‐control: *n* = 33; si‐MCU: *n* = 35). A Student's *t* test was used for statistical analyses. *p < 0.05; **p < 0.01; ns indicates nonsignificant (*p* > 0.05). Error bars show SEM

To further investigate MCU function, a specific siRNA microinjection procedure was used with fully grown oocytes at the GV stage. Firstly, we detected the interference efficient of the specific siRNA in mRNA and protein level (Figure S1A–D). The treated oocytes were arrested at the GV stage in medium with 2.5 μM milrinone for 20 h in order to consume endogenous MCU mRNA transcripts, followed by removal of milrinone and examination of meiotic maturation at different stages (Figure 2A). As expected, MCU knockdown caused a significant reduction in the proportion of GVBD oocytes (controls vs knockdowns, 86.33% vs. 62.30%, Figure 2B,C) and significantly reduced the proportion of MII oocytes (controls vs knockdowns, 73.87% vs.53.23%, Figure 2B,D). MCU defects increased the percentage of spindle defects in MI (controls vs. knockdowns, 6.7% vs.35.7%, Figure 3A,B) and MII (controls vs. knockdowns, 12.1% vs. 51.4%, Figure 3C,D). Additionally, Ru360, a specific inhibitor of MCU, was used to validate the siRNA results. As expected, result showed that the effect of Ru360 on meiotic progress and spindle assembly of oocytes was similar to knockdown of MCU with siRNA (Figure S2A–G). Together, these findings indicate that MCU is required for orderly oocyte meiotic maturation and that MCU knockdown oocytes are unable to properly assemble spindles during meiosis.

### 3.3 Mitochondrial Calcium uniporters is important for maintaining mitochondrial calcium homeostasis in mouse oocytes

Given that MCU had an important role in maintaining mitochondrial Ca^2+^, we stained the oocytes by Rhod‐2 AM and Flou‐3 AM to examine the [Ca^2+^]_m_ and [Ca^2+^] _i_. We found the level of [Ca^2+^]_m_ downregulated makeready but had no significant difference in [Ca^2+^]_i_ in MCU‐defected oocytes (Figure 2E–G). In additional, we treated oocytes by Ru360 to test [Ca^2+^]_m_ and [Ca^2+^]_i_ and we discovered Ru360 group has the same tendency as knockdown of MCU with siRNA (Figure S3A–F), whereas the MCU agonist spermine had the reverse effect of Ca^2+^ concentration change in oocytes (Figure S3G,H). Taken together, our data indicate that MCU played a key role in moderating mitochondrial calcium homeostasis.

### 3.4 Recognition of key effectors of si‐Mitochondrial Calcium Uniporters oocytes by single‐cell transcriptome analysis

**FIGURE 4:**
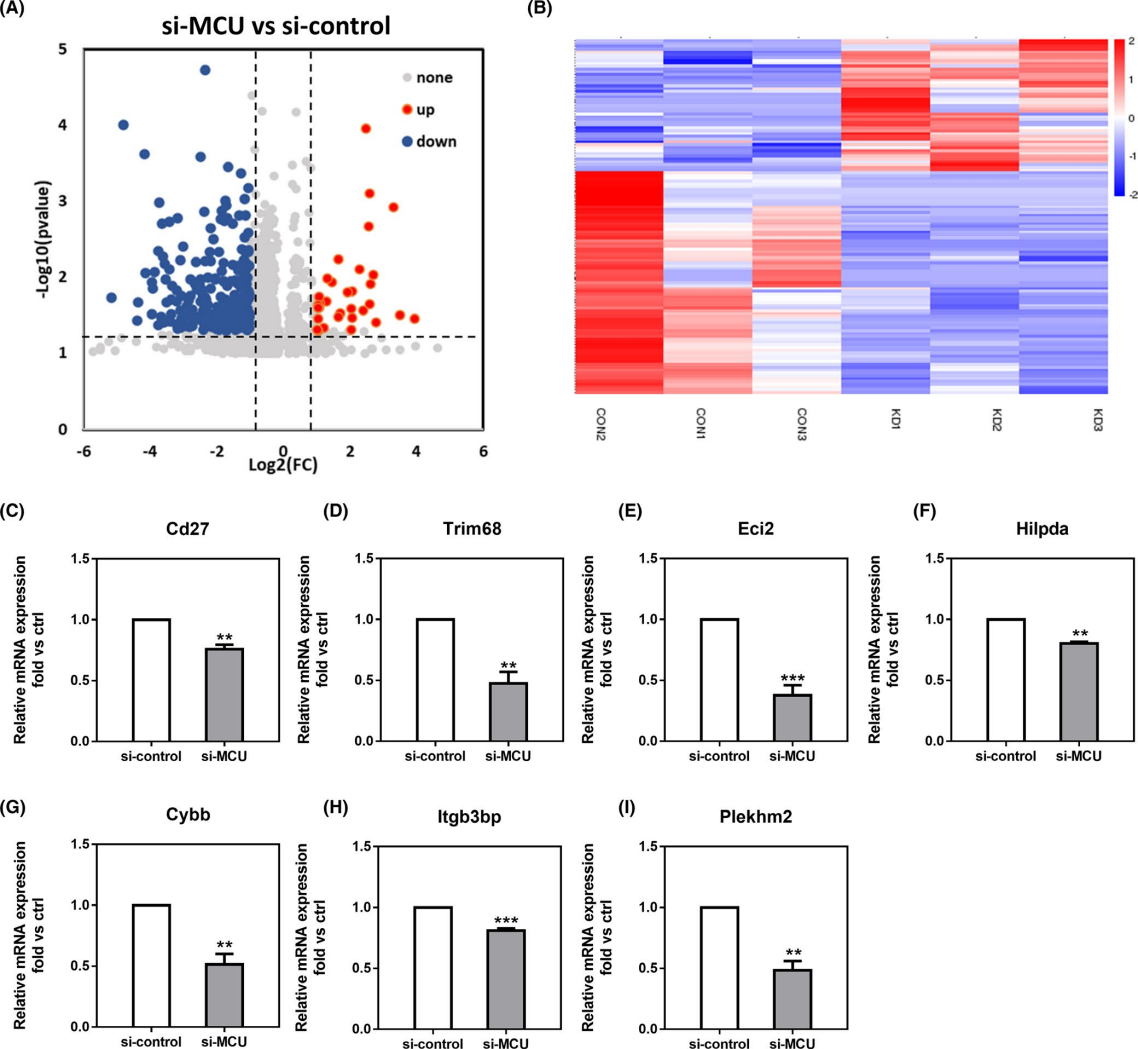
RNA‐seq date shows gene expression changes upon control and si‐MCU. (A) Volcano plot showing upregulated (red) and downregulated (green) genes in control and si‐MCU GV oocytes. (B) Heatmap of differentially expressed genes (DEGs) of control and si‐MCU GV oocytes. (C‐I) qRT‐PCR analyses of genes showed in the heatmap in control and si‐MCU GV oocytes. Results are presented as mean *± SEM*. **p < 0.01; ***p < 0.001

To delineate the genes and pathways affected in knockdown treatment on the quality of oocytes, we performed single‐cell transcriptome analysis of GV oocytes from control and si‐MCU. Volcano plot and heatmap data showed that the transcriptome profile of si‐MCU oocytes was obviously different from that of control oocytes, indicating that 47 differentially expressed genes (DEGs) were upregulated and 216 DEGs were downregulated in si‐MCU oocytes (Figure 4A,B). Furthermore, the expression of genes several randomly selected in twos groups was verified by quantitative real‐time PCR (Figure 4C–I).

**FIGURE 5:**
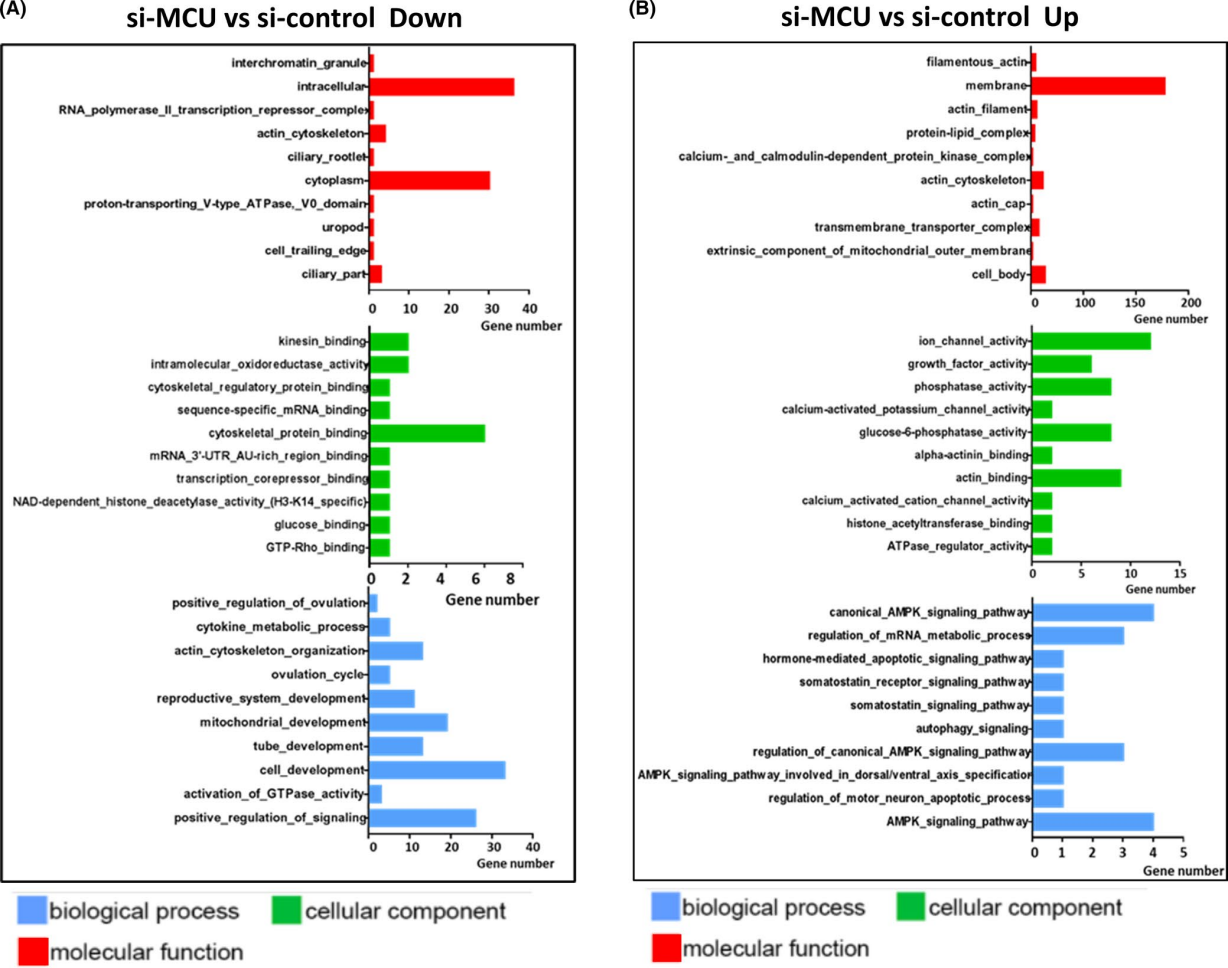
Effect of si‐MCU on transcriptome profiling of GV oocytes (A) GO enrichment analysis of downregulated DEGs in si‐MCU oocytes compared with control. (B) GO enrichment analysis of upregulated DEGs in si‐MCU oocytes compared with control. Blue represents biological processes, green represents cellular components, and red represents molecular function

As shown in Figure 5A,B, we used gene ontology (GO) analysis, showing that misregulated genes revealed overrepresentation of several biological processes. Especially in downregulated Go analysis, we found cytokine‐metabolic process changed evidently, which might decline energy synthesis in oocytes. Additionally, we noticed that reproduction development progress, actin‐cytoskeleton organization and mitochondrial development progress also had negative regulation, which indicated that some pivotal factor in oocytes quality had been damaged. Hence, all of these pathways or biological processes are highly related to energy metabolism, oxidative stress mitochondrial function, which prompts us to focus on mitochondria and energy sense reaction in si‐MCU oocytes.

### 3.5 Mitochondrial Calcium Uniporters is required for the maintenance of mitochondrial function and ATP content during oocyte meiosis

**FIGURE 6:**
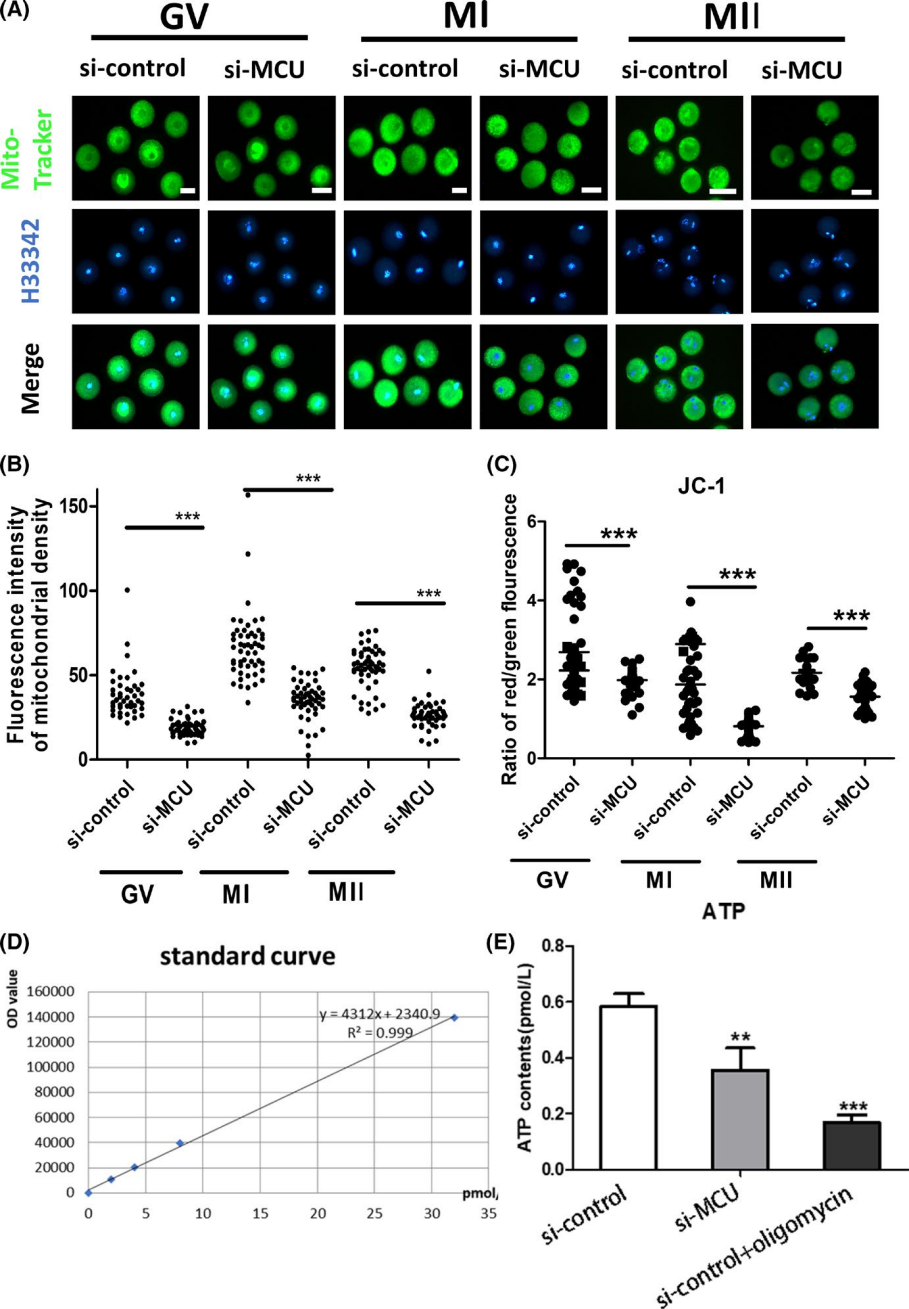
Mitochondrial function is impaired by si‐MCU (A) Representative images of Mito‐tracker (green) in si‐control and si‐MCU in GV, MI or MII oocytes. H33342 is shown in blue. Scale bar: 50 μM. (B) Quantification of the relative levels of mitochondrial masses in GV, MI or MII oocytes (*n* = 50 for each group). (C) si‐control and si‐MCU oocytes were stained with JC‐1 and subjected to quantification of the relative levels of mitochondrial membrane potentials (Δφm) in GV, MI or MII stages (*n* = 30 for each group). (D) ATP concentrations (pM) were measured in individual oocytes in the si‐control and si‐MCU groups (*n* = 30 for each group). (E) Standard curves were generated by serial dilution of known amounts of ATP standards to calculate ATP concentrations from the OD values of the samples. The correlation regression equation and coefficients (R^2^) are shown for ATP concentrations versus OD values. A Student's *t* test was used for statistical analyses. **p < 0.01; ***p < 0.001. Error bars show SEM

**FIGURE 7:**
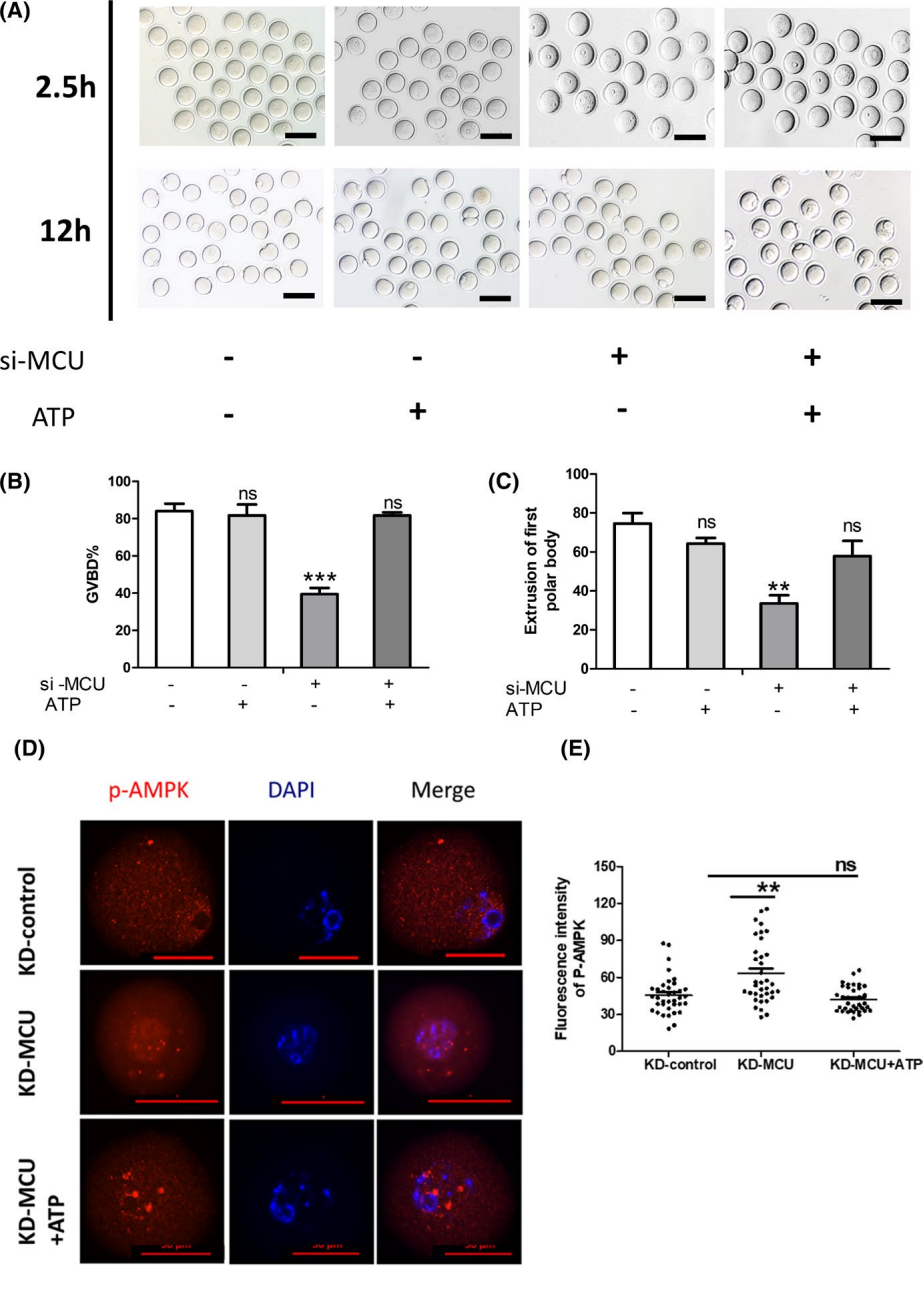
Mitochondrial function is impaired by si‐MCU (A) Representative images of germinal vesicle breakdown (GVBD) after 2.5 h and the first polar body (PB1) after 12 h for extrusion oocytes from the si‐control, si‐control +ATP, si‐MCU and si‐MCU + ATP treatments. Scale bar: 50 μM. (B) The percentage of oocytes that successfully progressed to GVBD during *in vitro* culture after 2.5 h (*n* = 119 for si‐control, *n* = 60 for si‐MCU, *n* = 77 for si‐control +ATP and *n* = 71 for si‐MCU + ATP). (C) The percentage of oocytes that successfully extracted the first polar body during *in vitro* culture after 12 h (*n* = 89 for si‐control, *n* = 61 for si‐MCU, *n* = 59 for si‐control +ATP and *n* = 92 for si‐MCU + ATP). (D) Confocal microscopy showing the subcellular localization and expression of p‐AMPK (red) in si‐control and si‐MCU groups in GV oocytes. DAPI staining is shown in blue. Scale bar: 20 μM. (E) Quantification of the relative levels of p‐AMPK in the si‐control, si‐MCU or si‐MCU + ATP oocyte groups (*n* = 37 for si‐control, *n* = 36 for si‐MCU and *n* = 37 for si‐MCU + ATP). A Student's *t* test was used for statistical analyses. **p < 0.01; ***p < 0.001. ns indicates nonsignificant (*p* > 0.05). Error bars show SEM

Since abnormal mitochondrial calcium signalling can significantly disturb the function of mitochondria, we examined the potential functions of MCU during oocyte meiosis. Fluorescently labelled mitochondria trackers were used to quantify the mitochondrial mass. Immunofluorescence observations demonstrated that si‐MCU adversely affects mitochondrial mass in GV, MI or MII oocytes (Figure 6A,B). Δφm values were then quantified using JC‐1, indicating that si‐MCU significantly decreased Δφm values in GV, MI or MII oocytes (Figure 6C). Accordingly, ATP levels in si‐MCU oocytes were significantly lower compared with controls (Figure 6D,E). To confirm that ATP contents are indispensable to meiosis progress, we then added 5 μM of exogenous ATP into the maturation solution and observed resumed meiosis in si‐MCU oocytes. After treatment of exogenous ATP, the meiotic defects caused by MCU knockdowns were distinctly ameliorated (Figure 7A–C). These observations confirmed that MCU regulates meiosis by maintaining normal mitochondrial dynamics and ATP levels.

### 3.6 Mitochondrial Calcium Uniporters knockdown activates the AMPK signal in mouse oocytes

**FIGURE 8:**
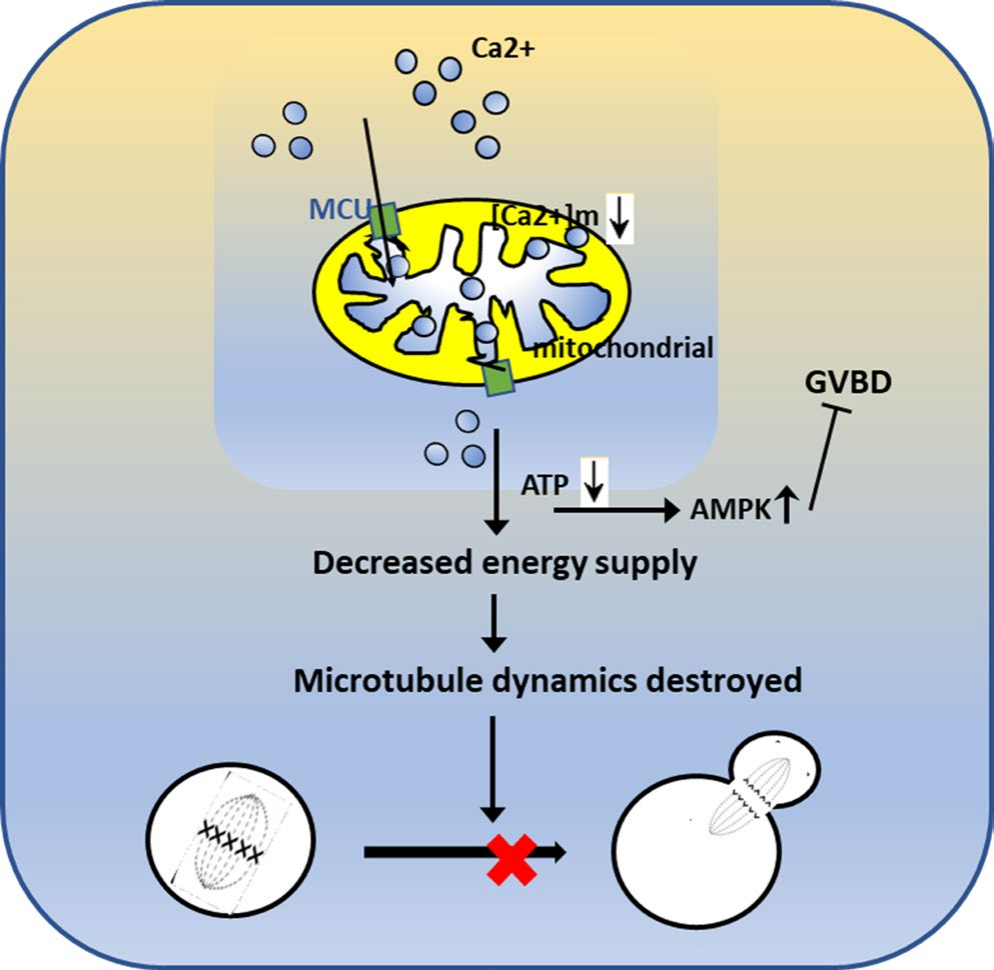
Model for MCU‐mediated ATP synthesis and abnormal AMPK activity in meiosis. When si‐MCU occurs in oocytes due to impaired mitochondrial function, cytosolic ATP levels decline. The energy sensor AMPK is activated and phosphorylated in response to the increased energetic stress. Excessive activation of AMPK results in adverse effects on the resumption of meiosis. In addition, microtubule dynamics and tension establishment cannot efficiently be achieved, leading to decreased meiotic progression. These observations all implicate MCU as being critical for meiotic progression

The effects of MCU defects on meiotic maturation and mitochondrial function led us to evaluate the potential mechanisms that would explain these phenotypes. From GO analysis, we noticed that some upregulated genes concentrated in AMPK signal (Figure 5B). Since a lower AMPK activity is activated by low ATP levels (Figure 7D,E), we expressed p‐AMPK in GV and MII oocytes by antibodies with immunofluorescence labels. Consistent with our hypothesis, immunofluorescence microscopy indicated that p‐AMPK exhibited significantly upregulated expression in oocytes after MCU depletion (Figure 7D,E). Si‐MCU oocytes at the GV stage were then treated with different concentrations of a distinct inhibitor of AMPK signal, Compound C. The inhibitor ameliorated meiotic maturation defects in MCU defection oocytes (Table 1). Collectively, these results suggest that MCU knockdown activates AMPK signalling in mouse oocytes and abnormally activated AMPK has universally adverse effects on meiosis progress (Figure 8).

## 4 DISCUSSION

Fully grown oocytes are rich with many kinds of maternal factors that are indispensable for meiotic maturation, fertilization and early embryonic development.^19^, ^20^ Furthermore, mitochondrially produced ATP acts as the basic energy source for many biological processes, since normal mitochondrial function plays a key role in maintaining ATP levels.^15^, ^21^, ^22^ After observing mitochondrial Ca^2+^ dynamics during different meiotic processes, we observed that changes in ATP content coincide with changes in mitochondrial calcium. We further demonstrated that mitochondrial calcium downregulates ATP production in mouse oocytes. Additionally, the mitochondrial Ca^2+^ uniporter (MCU) was identified as a major channel for Ca^2+^ uptake into the mitochondrial matrix. Numerous studies have suggested that MCU has a direct effect on mitochondrial calcium homeostasis.^23^ Mitochondrial Calcium uniporters is a multicomplex protein which consists of several proteins including the actual uniporter (MCU) and a series of regulators associated with the MCU. The regulatory molecules, which include the essential MCU regulator (EMRE), mitochondrial EF hand Ca^2+^ uniporter regulator (Micu1 and 2), are important in setting the threshold for Ca^2+^ uptake into the matrix.^24^, ^25^ Mitochondrial function is closely associated with mitochondrial calcium dynamics.^26^, ^27^ Accordingly, we pretreated si‐MCU GV oocytes with ATP and the meiotic delay was rescued, indicating that the loss of ATP supply is the main reason for the meiotic delay caused by MCU depletion.

Several studies have indicated that altered ATP levels can induce deleterious effects on normal cellular processes. Decreased production of mitochondrial ATP in oocytes may cause disassembly of MI and MII oocyte spindles.^28^ Based on these observations, we hypothesized that MCU depletion markedly disrupts spindle formation during oocyte meiosis. Many studies have shown that the segregation of sister chromatids during anaphase is a particularly important event in meiosis.^29^

In the previous study, some researchers have identified the importance of MCU in mitosis progression through regulating AMPK signal.^5^ AMPK is a heterotrimeric complex that comprises a catalytic α‐subunit and two regulatory subunits, β and γ.^30^ Recent studies have indicated that AMPK can sense glucose availability in addition to other metabolites in order to activate or inactivate pivotal metabolic pathways.^31^, ^32^ In addition to its roles in controlling various cellular activities, AMPK acts as a central integrator for regulating many kinds of mitochondrial functions.^33^ In addition, AMPK signalling has been widely confirmed to operate in oocyte maturation and that AMPK activity causes universally adverse effects on meiotic resumption.^32^, ^34^ However, little is still known about the relationship between AMPK and MCU during oocyte meiosis. Mammalian AMPK is thought to be activated by decreased cellular energy status, as signalled by changes in the ratios of ATP‐to‐ADP or ATP‐to‐AMP.^35^, ^36^ In this study, we hypothesized that MCU functions in oocyte meiotic maturation and is dependent on AMPK signalling pathways. Indeed, abnormal localization and expression of p‐AMPK after MCU defection was observed in GV and M II oocytes. MCU defection brought about impaired mitochondrial function, and cytosolic ATP levels decline in oocytes. The energy sensor AMPK is activated and phosphorylated in response to the increased energetic stress. Excessive activation of AMPK results in adverse effects on the resumption of meiosis. In addition, microtubule dynamics and tension establishment cannot efficiently be achieved, leading to decreased meiotic progression. Collectively, si‐MCU oocytes displayed a higher frequency of spindle defects in meiosis and affected oocyte maturation, possibly through changes in mitochondrial function and the p‐AMPK signalling pathway.

In conclusion, MCU was demonstrated here to be important in mouse oocyte maturation, and it is thereby a likely key regulator of mitochondrial ATP production and of important biological pathways including meiosis progress. MCU might modulate AMPK activity through these activities by controlling ATP contents. However, further studies are needed to confirm the precise mechanism and biological function by which MCU effects oocyte meiotic maturation.

## CONFLICT OF INTEREST

The authors declare no conflict of interest.

## AUTHOR CONTRIBUTIONS

Luyao Zhang participated in the research design, animal research, data analysis and writing of the paper; Lin Meng participated in animal research and writing of the paper; Luyao Zhang and Lin Meng contributed equally to the paper. Qing rui Zhuan, Jun jin Li, Ke xiong Liu and Zi chuan Wang participated in animal research, siRNA microinjection and revising of the paper. Xiangwei Fu participated in the writing and revising of the paper; Xiangwei Fu and Yun peng Hou provided substantial advice in designing the study and assisting in the division of labour, writing and revising the paper.

## Supporting information

Fig S1–S3

## Data Availability

The data that support the findings of this study are available from the corresponding author upon reasonable request.
