# Supplementary material for: Mitochondrial Calcium uniporters are essential for meiotic progression in mouse oocytes by controlling Ca2+ entry
Source: Cell Prolif. 2021 Sep 21;54(11):e13127. doi: 10.1111/cpr.13127 (PMC8560604; doi:10.1111/cpr.13127)
Supplement: Supplementary file 1 — Fig S1–S3 [file CPR-54-e13127-s001.docx]

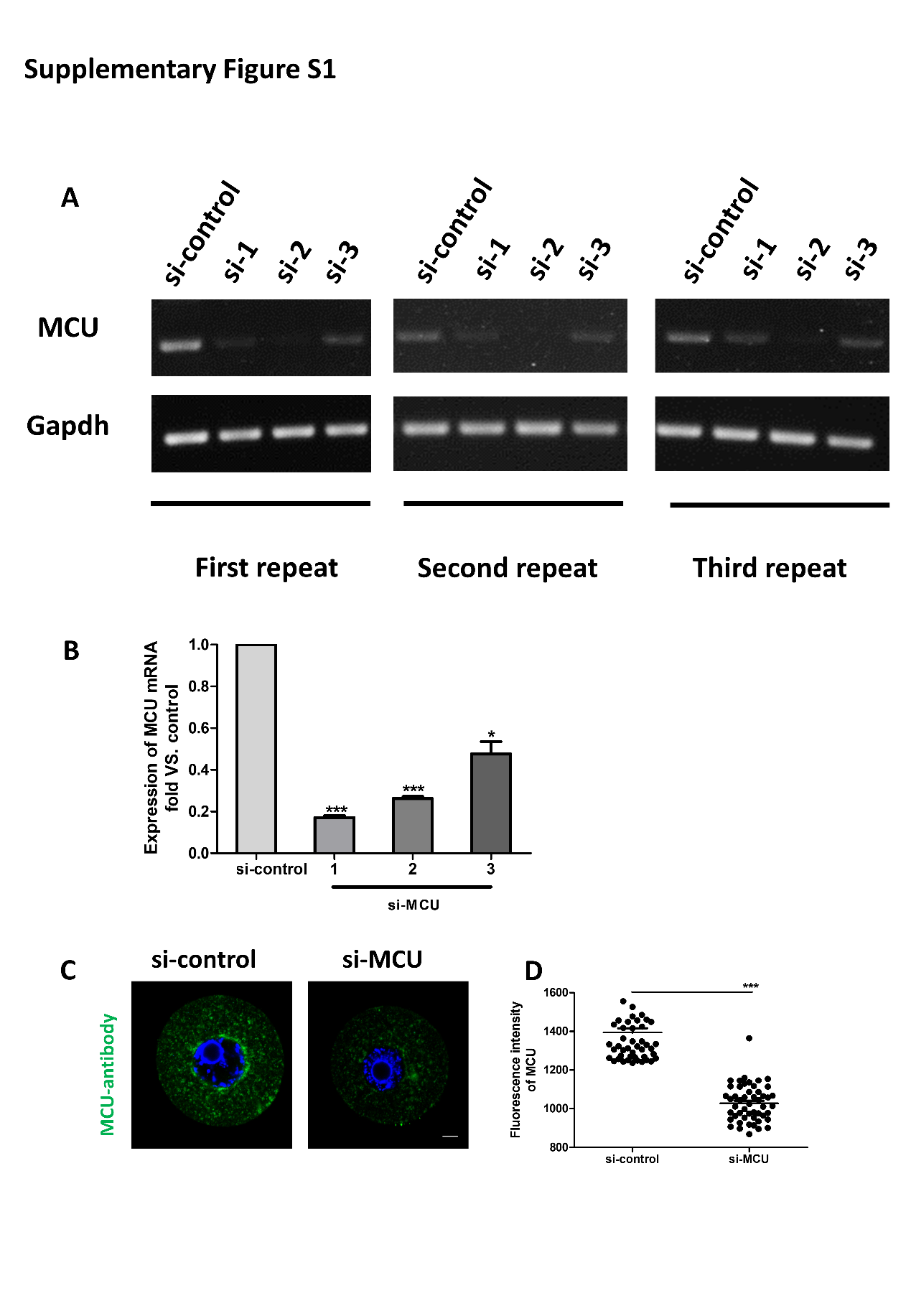


**Supplementary Figure S1.**

(A) The mRNA levels of MCU and Gapdh in control and three interference sequences of MCU oocytes, respectively (B)Quantitative Real-Time PCR showing MCU expression in control and three interference sequences of MCU oocytes, respectively. (n = 30 for each group) (C) Confocal microscopy showing the subcellular localization and expression of MCU (green) in si-control, and si-MCU groups in GV oocytes. DAPI staining is shown in blue. Scale bar: 20 μm. (D) Quantification of the relative levels of MCU in the si-control and si-MCU oocyte groups. (n = 50 for each group). Student’s *t-*test was utilized for statistical analyses. Error bars indicate mean ± SEM. * *P* < 0.05, *** *P* < 0.001 versus control group.


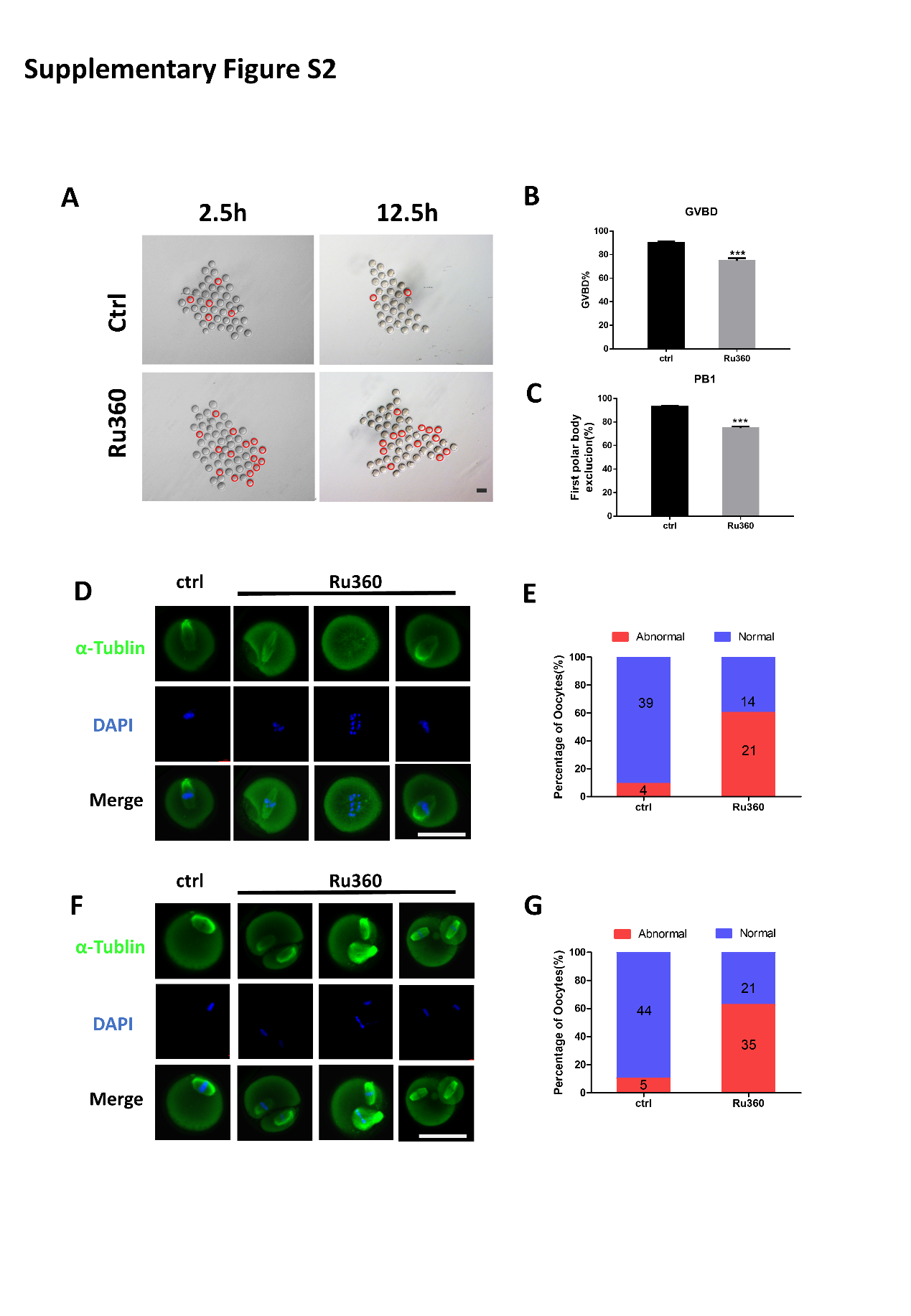


**Supplementary Figure S2.**

(A) Representative images of germinal vesicle breakdown (GVBD) after 2.5 h and the first polar body (PB1) after 12 h extrusion oocytes from the control and Ru360 groups. Scale bar: 50 μm. (B) The percentage of oocytes that successfully progressed to GVBD during in vitro culture for 2.5 h (n = 129 for control, n = 169 for Ru360). (C) The percentage of oocytes that successfully extracted the first polar body during in vitro culture for 12 h (n = 111 for control, n = 151 for Ru360). (D) Control(ctrl) and Ru360 M1 oocytes were co-stained with an α-tubulin antibody to visualize spindles (green) and with DAPI (blue) to visualize chromosomes. (E) Quantification of Control(ctrl) and Ru360 oocytes with abnormal spindles (ctrl: n = 43; Ru360: n = 35). (F) Control(ctrl) and Ru360 M2 oocytes were co-stained with an α-tubulin antibody to visualize spindles (green) and with DAPI (blue) to visualize chromosomes. (G) Quantification of Control(ctrl) and Ru360 M2 oocytes with abnormal spindles (ctrl: n = 49; Ru360: n = 56). A Student's t-test was used for statistical analyses. ***: p < 0.001. Error bars show SEM.


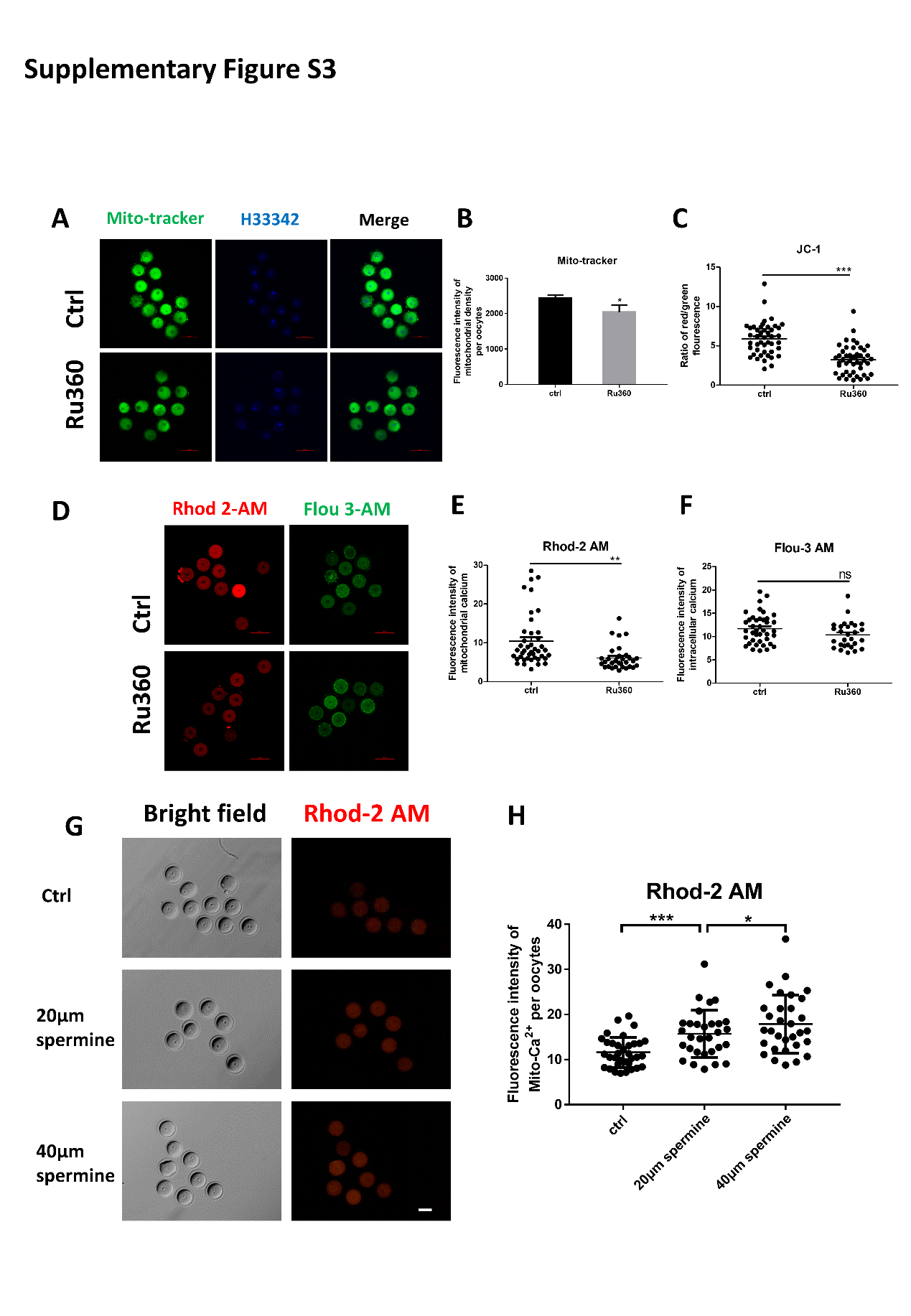


**Supplementary Figure S3.**

(A) Representative images of Mito-tracker (green) in control and Ru360 oocytes. H33342 is shown in blue. Scale bar: 50 μm. (B) Quantification of the relative levels of mitochondrial masses in control and Ru360 oocytes. (n = 50 for each group). (C)Control and Ru360 oocytes were stained with JC-1 and subjected to quantification of the relative levels of mitochondrial membrane potentials (Δφm) (ctrl: n = 45; Ru360: n = 47). (D) Representative images of Rhod-2 AM fluorescence (red) and Flou-3 AM fluorescence(green) in germinal vesicle (GV) stage oocytes from the control and Ru360 oocytes. Scale bar: 50 μm. (E) Quantification of the relative levels of mitochondrial Ca^2+^ in GV-stage oocytes from control and Ru360 group. (ctrl: n = 42; Ru360: n = 32). (F) Quantification of the relative levels of cytoplasmic Ca^2+^ in GV-stage oocytes from control and Ru360 group. (n = 28 for each group). (G) Representative images of Rhod-2 AM fluorescence (red) in germinal vesicle (GV) stage oocytes from the control ,20μm and 40μm spermine oocytes. Scale bar: 50 μm. (H) Quantification of the relative levels of mitochondrial Ca^2+^ in GV-stage oocytes from the control ,20μm and 40μm spermine oocytes. (ctrl: n = 34; 20μm spermine=30 ;40μm spermine: n = 31).

A Student’s t-test was used for statistical analyses. *: p < 0.05; **: p < 0.01; ***: p < 0.001; ns. indicates non-significant (p > 0.05). Error bars show SEM.
